# *Helicobacter pylori* Infection and Autoimmune Thyroid Diseases: The Role of Virulent Strains

**DOI:** 10.3390/antibiotics9010012

**Published:** 2019-12-30

**Authors:** Natale Figura, Giovanni Di Cairano, Elena Moretti, Francesca Iacoponi, Annalisa Santucci, Giulia Bernardini, Stefano Gonnelli, Nicola Giordano, Antonio Ponzetto

**Affiliations:** 1 Department of Biotechnology, Chemistry and Pharmacy, University of Siena, 53100 Siena, Italy; natale.figura@unisi.it (N.F.); annalisa.santucci@unisi.it (A.S.); 2 Department of Medical, Surgical and Neurological Sciences, University of Siena, 53100 Siena, Italy; giovanni.dicairano@unisi.it (G.D.C.); stefano.gonnelli@unisi.it (S.G.); nicola.giordano@unisi.it (N.G.); 3 Department of Molecular and Developmental Medicine, University of Siena, 53100 Siena, Italy; elena.moretti@unisi.it; 4 Department of Food Safety and Veterinary Public Health, National Institute of Health, 00161 Rome, Italy; francesca.iacoponi@iss.it; 5 Department of Medical Sciences, University of Torino, 10126 Torino, Italy; ponzettoa@yahoo.it

**Keywords:** *H. pylori* infection, CagA virulence factor, autoimmune thyroid diseases, Greaves’ disease, Hashimoto thyroiditis, anti-thyroglobulin autoantibodies, inflammatory cytokines, antigenic mimicry

## Abstract

Aim: To verify a possible association between overall *H. pylori* and CagA+ *H. pylori* infection and autoimmune thyroid diseases (AITDs). Methods: Consecutive patients with AITDs admitted to one single centre of Endocrinology during one solar year were examined. The diagnoses were Hashimoto thyroiditis (HT) in 76, Graves’ Disease (GD) in 39, and aspecific thyroiditis (AT) in 44 patients. Controls were 136 individuals without AITDs. Median values of fT3, fT4, anti-thyreoglobulin (Tg) antibodies, IL-1β, IL-6, and TNF-α in patients were compared with those in controls. *H. pylori* infection and CagA status were determined serologically. Structural homology of some thyroid proteins with *H. pylori* antigens was investigated. Results: *H. pylori* infection prevalence was significantly increased in GD (66.6%) and HT (64.4%) patients, vs. 29.4% of controls and 34.0% of AT. CagA seropositivity was significantly more frequent in GD (46.1%) and HT (46.9%) infected patients, vs. infected controls (20%). fT3 and fT4 median values were significantly decreased in infected CagA+ GD patients vs. uninfected GD patients. IL-1β median values were increased in patients respect to controls, independently of the clinical form of AITD. Median values of IL-6, TNF-α and anti-Tg autoantibodies in CagA infected patients were significantly higher than those measured in infected CagA− and uninfected patients and in infected CagA+ controls. The examined thyroid proteins shared putative conserved domains with numerous bacterial antigens. Conclusions: Overall *H. pylori* and CagA+ *H. pylori* infection were associated with GD and HT, putatively through an increased inflammatory status and molecular mimicry.

## 1. Introduction

Autoimmune disorders (ADs) are very frequent and encompass a broad variety of diseases [1,2,3]. The organ most frequently involved in ADs, either independently of, or in concomitance with other organs, is the thyroid [4,5,6]. The spectrum of autoimmune thyroid disorders (AITDs) is wide and encompasses (a) aspecific thyroiditis (AT), where the organ functionality is maintained but it is heavily infiltrated by lymphocytes; (b) Hashimoto thyroiditis (HT), characterized by hypothyroidism; or (c) Graves’ (GD) or Basedow disease, characterized by hyperthyroidism [6]. These three different functional conditions affect 2–5% of the general population, but they occur far more frequently in people with the human leucocyte histocompatibility antigen HLA-DRB1*03 [7].

AITDs originate from an autoimmune response against gland antigens: The thyroid peroxidase (TPO), thyroglobulin (Tg) and the thyroid stimulating hormone receptor (TSHr). Two-thirds of HT patients and one third of those with GD possess circulating anti-Tg antibodies. Anti-TPO antibodies circulate in 90% ca. of patients with HT and 75% ca. of those with GD [8,9]. Being the hallmark of GD, the presence of agonistic anti-TSHr antibodies concerns nearly all cases of this pathology [10].

*Helicobacter pylori* infection would be the ideal candidate for the role of one of the causative agents in the development of AITDs [11]: First of all, (a) *H. pylori* infection is a condition in which autoimmunity is exalted [12]; (b) both the stomach and thyroid originate from the foregut [13]; and (c) in cases of autoimmune thyroiditis, lymphocytes infiltrating the thyroid are organized as lymphoid tissue [14], as observed in the gastric mucosa of *H. pylori* infected people; (d) sometimes, thyroid lymphoid tissue shares features of gastric mucosal-associated lymphoid tissue (MALT), a condition linked to *H. pylori* infection [13]; (e) in 1997, Tomb et al. described the presence of a gene encoding an endogenous peroxidase in the dissected chromosome of an *H. pylori* strain [15], finally; (f) there is a cross-reactivity between anti-*H. pylori* monoclonal antibodies and thyroid follicles, suggesting the existence of antigenic mimicry phenomena between thyroid and bacterial structures [16].

In a previous study, we observed that serum antibodies to *H. pylori* cytotoxin-associated gene A product, CagA, were significantly more prevalent in patients than in controls [17]. CagA-positive strains are endowed with an enhanced inflammatory potential and in infected people they increase the gastric and systemic levels of inflammatory cytokines [18,19,20,21]. Neutrophil and basophil blood counts are also augmented in infected CagA-positive patients [22] and, together with cytokines and autoantibodies, may concur to exacerbate and even promote the inflammatory aggression of thyroid [23].

There is not a full consensus on the possible association between *H. pylori* infection and AITDs, partially explained by the incomplete characterization of the infecting organisms (i.e., whether they are CagA-positive or -negative) and by the fact that the different clinical forms of AITDs are considered all together rather than as separate entities [24,25].

The aim of the present study was to investigate the possible role of *H. pylori* infection, especially by strains expressing CagA, in the development of AITDs. We compared the circulating levels of thyroid hormones, thyroid autoantibodies and some inflammatory cytokines in infected patients with those determined in uninfected patients and in controls without AITDs. Finally, to substantiate the possible role of antigenic mimicry phenomena in the development of thyroid autoantibodies, we aligned Tg, TPO and TSHr with *H. pylori* peptides to verify the existence of areas of homologies between human and bacterial antigens.

## 2. Results

### 2.1. Prevalence of Overall H. pylori and CagA Positive H. pylori Infection

The percentage of *H. pylori* infection and the CagA status in patients and controls was reported in Table 1.

P values and ORs concerning the prevalence differences between patients and controls are reported in Table 2. Briefly, infection by *H. pylori* in GD and HT patients was significantly more prevalent than in controls (*p* < 0.001) and in patients with AT (*p* < 0.01 and *p* < 0.001, respectively). Similar results were obtained as far as the infection by strains expressing CagA was concerned: GD vs. controls, *p* < 0.05; HT vs. controls, *p* < 0.01. No other difference was statistically significant.

### 2.2. Levels of Thyroid Hormones

**Figure 1:**
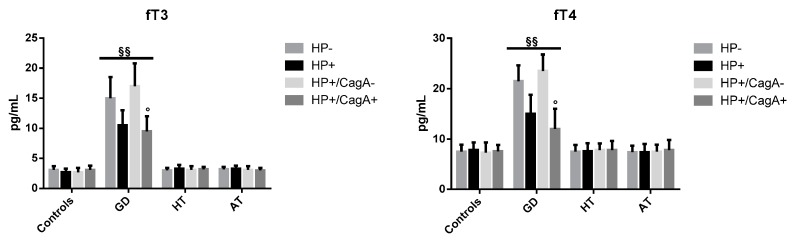
Median (IQR) values of fT3 (**left** and fT4 (**right**) in patients with different clinical forms of AITD and controls according to the overall *H. pylori* and CagA infectious status. ° *p* < 0.05: HP+/CagA+ vs. HP+/CagA−, §§ *p* < 0.01 AITDs vs. no-AITDs.

The median values (IQR) of fT3 are shown in Figure 1 (left panel). Levels of fT3 in infected GD patients were decreased with respect to those observed in uninfected GD patients (10.5 (2.5) vs. 15.0 (3.5) pg/mL), but the difference was not significant. CagA seropositive patients had median fT3 values significantly lower than had the CagA seronegative patients (9.5 (2.5) vs. 17 (3.8) pg/mL, *p* < 0.05). The fT3 median values (IQR) in HT patients were 3.3 (0.6), 3.0 (0.4), 3.1 (0.4), and 3.4 (0.5) pg/mL, respectively in HP+, HP−, HP+/CagA+, and HP+/CagA− patients. Differences were not significant. In AT patients, levels were 3.3 (0.5), 3.2 (0.4), 3.0 (0.4), and 3.1 (0.6) pg/mL, respectively in HP+, HP−, HP+/CagA+, and HP+/CagA− patients. Differences were not significant. Finally, in controls, fT3 median values (IQR) were 2.7 (0.6), 3.1 (0.6), 3.1 (0.7), and 2.7 (0.7) pg/mL. Differences were not significant.

The median values (IQR) of fT4 are shown in Figure 1 (right panel). In infected GD patients, fT4 levels were lower than those observed in uninfected GD patients (15.0 (3.8) vs. 21.5 (3.1) pg/mL) but the difference was not significant. CagA seropositive patients had median fT4 values significantly lower than had the CagA seronegative patients (12.0 (4.0) vs. 23.5 (3.3) pg/mL, *p* < 0.05). In HT patients, median fT4 values were 7.6 (1.6), 7.6 (1.6), 7.8 (1.8), and 7.8 (1.3) pg/mL, respectively in in HP+, HP−, HP+/CagA+, and HP+/CagA− patients. Median fT4 serum concentrations in AT patients 7.4 (1.6), 7.4 (1.3), 7.8 (2.0) and 7.5 (1.4) pg/mL, respectively in HP+, HP−, HP+/CagA+ and HP+/CagA− patients. In controls, the median fT4 values were 7.8 (1.5), 7.5 (1.4), 7.6 (1.2), and 7.3 (2.0) pg/mL, respectively in HP+, HP−, HP+/CagA+, and HP+/CagA− patients.

Levels of fT3 and fT4 in infected and uninfected GD patients were higher, as expected, than those measured in infected and uninfected controls without AITDs (*p* < 0.01).

### 2.3. Levels of Anti-Tg Autoantibodies

**Figure 2:**
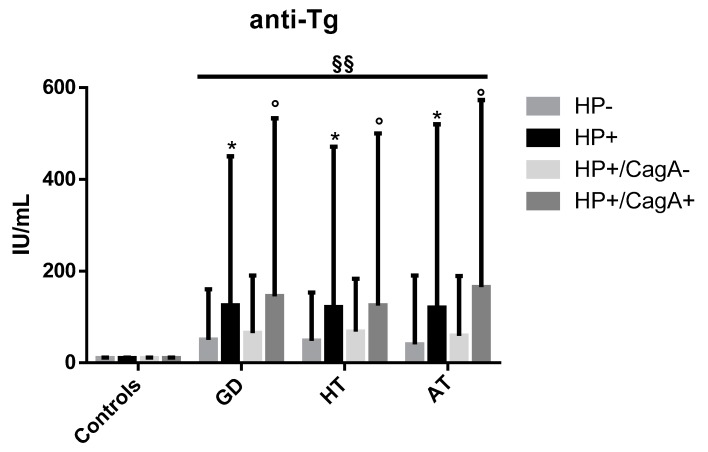
Median values (IQR) of anti-Tg serum antibodies in patients and controls according the overall *H. pylori* infectious and CagA status. * *p* < 0.05: HP+ vs. HP− patients, ° *p* < 0.05: HP+/CagA+ vs. HP+/CagA− patients, §§ *p* < 0.01 AITDs vs. no-AITD.

These results are reported in Figure 2. In patients with GD, the anti-Tg median antibodies (IQR) values were 125 (375), 50 (110), 145 (388), and 65 (125) IU/mL, respectively in HP+, HP−, HP+/CagA+, and HP+/CagA− patients. In HT patients, median values were 121 (350), 48 (105), 125 (375), and 68 (115) IU/mL, respectively in HP+, HP−, HP+/CagA+, and HP+/CagA− patients. In AT patients, median values were 120 (400), 40 (150), 165 (408), and 59 (130) IU/mL, respectively in HP+, HP−, HP+/CagA+, and HP+/CagA− patients. Controls, as a rule, did not have autoantibodies. Anti-Tg autoantibodies were significantly more prevalent in infected patients with respect to the uninfected ones (*p* < 0.05), and in CagA seropositive patients with respect to patients who did not have serum antibodies to CagA (*p* < 0.05).

### 2.4. Levels of Inflammatory Cytokines

The median values (IQR) of IL-1β were 0.2 (0.2) in all patients, independently of the infectious and CagA status (excepted in uninfected AT patients in whom the median value was 0.3 (0.2)). Mean values were 0.1 (0.1) in all controls (data not shown). IL-1β levels in patients, independently of the infectious and the CagA status, were increased respect to those detected in both infected and uninfected controls (*p* < 0.05).

**Figure 3:**
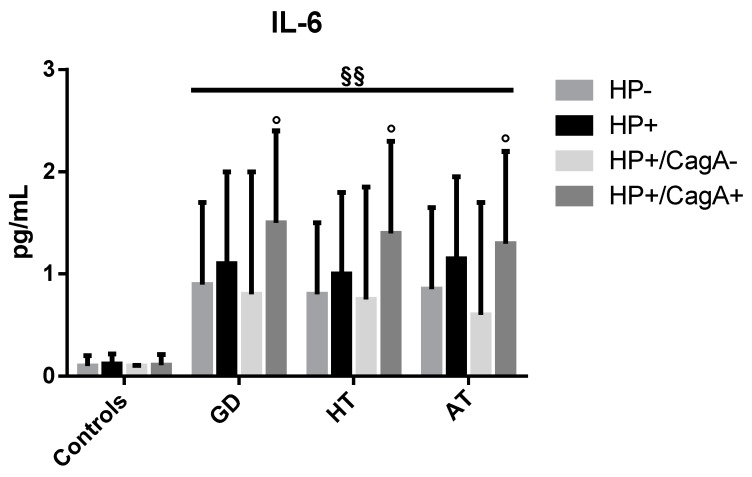
Median values (IQR) of IL-6 in patients and controls according to the overall *H. pylori* and CagA infectious status. ° *p* < 0.05: HP+/CagA+ vs. HP+/CagA− patients, §§ *p* < 0.01 AITDs vs. no-AITD.

The median values (IQR) of IL-6 are reported in Figure 3. In GD patients, median values were 1.1 (0.9), 0.9 (0.8), 1.5 (0.9), and 0.8 (1.2) pg/mL, respectively in HP+, HP−, HP+/CagA+, and HP+/CagA− patients. In HT patients, median values were 1.0 (0.8), 0.8 (0.7), 1.4 (0.9), and 0.75 (1.1) pg/mL, respectively in HP+, HP−, HP+/CagA+, and HP+/CagA− patients. In AT patients, median IL-6 values were 1.15 (0.8), 0.85 (0.8), 1.3 (0.9), and 0.6 (1.1) pg/mL, respectively in HP+, HP−, HP+/CagA+, and HP+/CagA− patients. In controls, median levels were 0.12 (0.1), 0.10 (0.1), 0.11 (0.1), and 0.10 (0.007) pg/mL, respectively in HP+, HP−, HP+/CagA+, and HP+/CagA− patients. In AITDs patients, IL-6 levels were higher in infected than in uninfected individuals, and in CagA-positive than in infected CagA-negative patients (*p* < 0.05). IL-6 in infected and uninfected AITDs patients was higher than controls (*p* < 0.01), independent of the infectious and CagA status.

**Figure 4:**
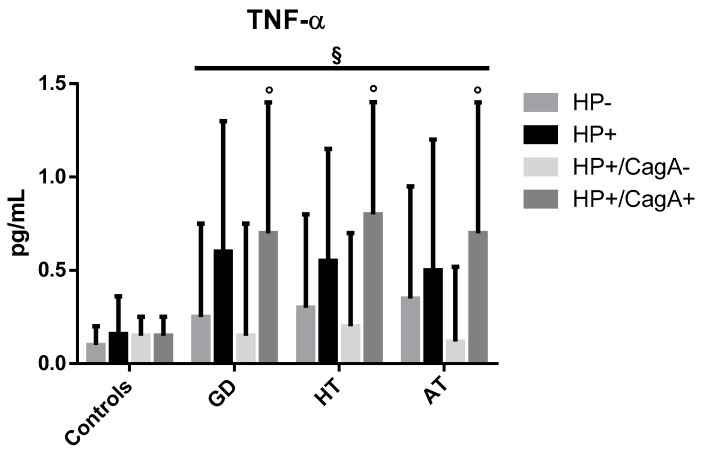
Median values (IQR) of TNF-α in patients and controls according to the overall *H. pylori* and the CagA infectious status. ° *p* < 0.05: HP+/CagA+ vs. HP+/CagA− patients, § *p* < 0.05 AITDs vs. no-AITD.

The median values (IQR) of TNF-α are reported in Figure 4. In GD patients, median values were 0.6 (0.7), 0.25 (0.5), 0.7 (0.7), and 0.15 (0.6) pg/mL, respectively in HP+, HP−, HP+/CagA+, and HP+/CagA− patients. In HT patients, values were 0.55 (0.6), 0.3 (0.5), 0.8 (0.6), and 0.2 (0.5) pg/mL, respectively in HP+, HP−, HP+/CagA+, and HP+/CagA− patients. In AT patients, TNF-α mean values were 0.5 (0.7), 0.35 (0.6), 0.7 (0.7), and 0.12 (0.4) pg/mL, respectively in HP+, HP−, HP+/CagA+ and HP+/CagA− patients. In controls, mean values were 0.16 (0.2), 0.10 (0.1), 0.15 (0.1), and 0.15 (0.1) pg/mL, respectively in HP+, HP−, HP+/CagA+, and HP+/CagA− patients. In AITDs patients, TNF-α levels were significantly higher in CagA-positive than in infected CagA-negative patients (*p* < 0.05). TNF-α in infected and uninfected AITDs patients was higher than controls (*p* < 0.01), independent of the infectious and CagA status.

### 2.5. Alignments

**Figure 5:**
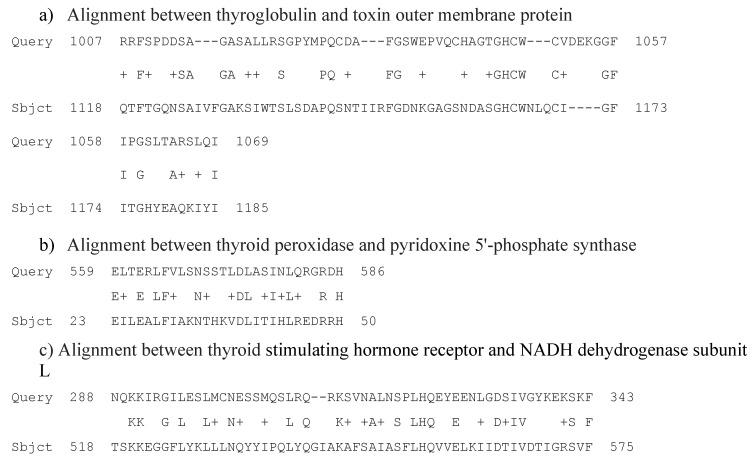
Main putative conserved domains detected by aligning thyroglobulin, thyroid peroxidase, and thyroid stimulating hormone receptor with *H. pylori* proteins. “Query”: Amino acid sequence of the human proteins. “Subject”: Amino acid sequence of the bacterial protein present in the data base. “+” indicates that the amino acid in the sequence “query” and that one in the sequence “subject” are similar from a chemical-physic point of view. The numbers in the sequence represent the initial and final positions of the amino acid sequence.

Thyroglobulin shared putative conserved domains with three bacterial proteins: Toxin outer membrane protein (also reported in Table 3 and Figure 5), GTP-binding protein, and type II restriction endonuclease.

Thyroid peroxidase showed a partial linear homology with five bacterial proteins: Pyridoxine 5′-phosphate synthase (Table 3 and Figure 5), peptidase M23, a hypothetical protein, ribonuclease R, and sodium-calcium antiporter.

Thyroid stimulating hormone receptor partly aligned with nine bacterial proteins: NADH dehydrogenase subunit L (Table 3 and Figure 5), trigger factor, Sel1 repeat protein HcpA, DNA-binding protein, RNA degradosome polyphosphate kinase, hypothetical protein HPG27_109, ABC transporter, permease, radical SAM protein, and hypothetical protein HPG27_1376.

## 3. Discussion

The most relevant message from our study is that patients suffering from overt AITDs were more frequently infected by *H. pylori* than controls, i.e., individuals without thyroid inflammatory involvement. In particular, an increased prevalence of infection was found in patients affected with GD, as already observed by other authors [26], and HT. Patients with AT, on the contrary, were infected with a frequency similar to controls. The low prevalence of *H. pylori* infection in patients with AT, although surprising, may simply reflect an early stage of the disease. We hypothesized that patients with AT represent a small fraction of individuals who experienced *H. pylori* infection, able to trigger an inflammatory thyroid disorder, whose immune response, and possibly other factors, have concurred to eradicate the infection. Spontaneous eradication of infection, in fact, is not a rare event. In adults, over an interval of 3–32 years, sero-reversion concerned 6.04% of 1806 individuals. In childhood, sero-reversion is even far more common, reaching figures as high as 41% of subjects [27]. In such patients, thyroiditis does not progress towards GD or HT, but persists in the form of AT, for different and so far unknown reasons, possibly including the resolution of *H. pylori*-related systemic inflammation.

De Groot et al. [28] presented the development of overt AITDs in six steps, each of whom could be possibly reverted. They hypothesized that the initial damage could be caused by infections in genetically susceptible individuals carrying the necessary HLA DR and DQ antigens. The second step could be regression or progression, depending on general inflammatory conditions. All the following steps rely on variability of complement factors, genetics, NK cells, and/or macrophage activation, reactivation, or deactivation. Only when all these conditions are satisfied and reach a point of no return, will the clinical symptoms appear. An example of this balance of factors is the so-called “adolescent goiter”, an enlargement of the gland during the second decade accompanied by normal function tests with thyroid autoantibodies. If a biopsy is performed, the diagnosis is HT, but the regression is the rule, as age goes on [29].

The results of the present study have also shown that the expression of CagA by the infecting organisms may play an important additional role in the development or worsening of AITDs, despite there is not a complete agreement on this point [30]. Recent meta-analyses have shown that the presence of anti-CagA antibodies in the blood of *H. pylori* infected patients enhances the risk for AITDs [31,32]. Infection by CagA-positive organisms may play a critical role in the development of multifactorial diseases such as AITDs. This conclusion was reached by our group in a cohort of female patients: The frequency of infection in patients and controls was almost the same, but anti-CagA serum antibodies were significantly more prevalent in patients [17]. Such protein is the marker of virulent strains and individuals infected by these organisms experience an elevated local and systemic inflammatory status, which may concur to promote the clinical manifestation of those diseases, such as AITDs, in which inflammation plays an important role.

One of the most important constituents of inflammation is cytokine production. The blood levels of IL-6 among *H. pylori*-positive patients were almost ten times higher than among controls, and (which it is even more interesting), among CagA-positive patients, they were twice as high than in infected CagA-negative patients. Also, the median values of TNF-α reached strikingly higher levels in CagA-positive AITD patients compared to the negative ones.

This increased secretion of proinflammatory cytokines in CagA-positive AIDTs patients strongly reinforce the idea that pathogenic strains of *H. pylori* might initiate or amplify (or both) the autoimmune response [18,19,20,33]. The observation that serum levels of IL-1β were not increased in infected patients is not unusual. Mehmet et al. [34] found that all cytokines were increased in gastric fluids and sera of infected gastric cancer patients, with the exception of IL-1β,. In that study, the genotype of strains was not characterized, however, we can presume that organisms associated with gastric cancer were in great majority CagA-positive. Thus, despite the fact that IL-1β is a proinflammatory cytokine, it may not increase systemically in *H. pylori*-infected individuals. In HT, cytokine response is most probably responsible for destroying thyrocytes, mostly via T cell-mediated killing, causing reduced levels of fT3 and fT4. On the contrary, the circulating levels of these hormones in GD patients were very elevated as expected. At first, however, we found unusual that CagA-positive patients with GD had lower fT3 levels compared to both CagA-negative infected patients and CagA-positive infected controls. We argued that the extremely elevated concentrations of serum proinflammatory cytokines could explain, at least in part, this finding. Levels of IL-6 and TNF-α, in fact, were extremely increased among CagA-positive patients compared to CagA-negative ones, and it is well established that inflammatory cytokines do lower the level of thyroid hormones acting directly on cell functions [35]. Moreover, cytokines were shown to determine the initiation of thyrocyte death by activating Fas-dependent cell destruction. The exposure of thyroid cell cultures to gamma-interferon and TNF-α determined in vitro a rapid and severe Fas-mediated apoptosis, even though in GD the infiltrating lymphocytes generally are not destructive as they are in HT [36].

Other researchers reported reduced concentrations of thyroid hormones, as well as high titers of thyroid autoantibodies, in *H. pylori*-infected blood donors [37]. Despite the fact that these authors did not characterize the examined infected individuals for the presence of serum antibodies to CagA, the results of Triantafillidis et al.’s study suggest that many determinants may contribute to producing them (environmental factors, such as smoking and alcohol consumption, genetic predisposition, etc.). It seems therefore reasonable to add to these factors a test to diagnose the CagA status.

The thyroid antigens most frequently used to diagnose and follow the AITDs are Tg, TPO, and TSH-R. We hypothesized that these antigens could share a structural homology with bacterial proteins. We therefore compared the sequences of the three antigens with those from a *H. pylori* strain isolated in the same region where patients lived, strain G27. The molecular mimicry exhibited by Tg, TPO, and TSH-R was noteworthy for respectively three, five, and nine different bacterial proteins, and likely sufficient to break immune tolerance for autoantigens [38]. Another possible reason for the breaking of immune tolerance is the presentation of antigens at an unusual location, as is the case for bacteria encountering immune cells in the Peyer’s patches of the intestine. Arguably, the autoantibodies against these mimicked antigens should be found at a high frequency in the population, which is the case: Up to 36% of “normal” female adults were found to carry some levels of anti-thyroid autoantibodies [28].

Titers of anti-Tg autoantibodies were steadily increased in patients carrying CagA expressing strains both in GD and in HT patients. The presence of anti-Tg autoantibodies is a common feature in AITD patients, independently of *H. pylori* infection, which therefore is neither a necessary nor a sufficient condition for they production. A recent study has shown that the median levels of anti-*H. pylori* serum antibodies were increased in children infected by strains expressing CagA [39]. It is known that gastric colonization by strains expressing CagA stimulates the infiltration and activation of polymorphs and lymphocytes into the gastric epithelium through the secretion of IL-6, IL-8, and other proinflammatory cytokines [40,41,42]. A greater number of lymphocytes could correspond to higher levels of antibodies and autoantibodies, too. In addition, it has recently been shown that CagA can also be translocated into human B lymphocytes through the conjugative apparatus set up by *cag* genes upstream *cagA* [41]. After cytoplasm translation, CagA may exert many activities, including the upregulation of the anti-apoptotic proteins Bcl-2 and Bcl-X in human B lymphocytes. As a consequence, lymphocyte apoptosis is inhibited, and cell replication is stimulated. Such phenomenon could concur to determine MALT formation [43] and most probably determine an enhanced production of antibodies by the stimulated lymphocytes. In conclusion, whatever it is the antigen that stimulates the secretion of autoantibodies in AITD patients, the presence of the CagA antigen may be a main factor that affects the levels of autoantibodies.

## 4. Materials and Methods 

### 4.1. Patients and Controls

We carried out a case-control study on 159 patients (mean age 52 years, range 15–79 years) attending the Endocrinology Unit of Siena University Hospital from the 1st of January to the 31st of December 2009: 76 patients with HT (almost all in substitutive treatment), 39 patients with GD (all treated with methimazole, with a few exceptions), and 44 patients with AT. The diagnosis of AITD was based on clinical examination, thyroid ultrasounds, titration of thyroid hormones and thyroid autoantibodies (anti-TPO and anti-TSH-R). Other relevant co-morbidities were not present. The control group was composed of 136 subjects with no concomitant known autoimmune/inflammatory conditions, comparable for age (mean age 53 years, range 18–86 years) and gender. Samples were collected at Siena University Hospital. Patients and controls gave their informed consent in writing and the study was approved by the Hospital’s Ethical Committee.

Blood samples were collected from a cubital vein between 9:00 and 10:30 a.m. after 12 h of fasting and were drawn in color-coded Vacutainer^®^ tubes (BD). Blood was allowed to clot for 60 min ca.; serum was separated by centrifugation at 4 °C and stored at −20 °C for the determination of *H. pylori* infectious and CagA status and the levels of thyroid autoantibodies. Part of the serum samples was stored at −80 °C until inflammatory cytokine levels were assayed.

### 4.2. Determination of Thyroid Hormones

Thyroid hormones levels were assessed the same day of blood collection. Serum specimens were used to determine the levels of free triiodothyronine (fT3) by fluorescence enzymatic method ELFA (BioMérieux, Marcy-l’Étoile, France), free thyroxine (fT4) and thyroid stimulating hormone (TSH, only to determine the diagnosis) by chemiluminescent immunoassay (Immulite, Diagnostic Product Corporation, Los Angeles, CA, USA). Results were expressed in pg/mL, as reported in the manufacturer’s instructions.

### 4.3. Determination of Thyroid Autoantibodies

Levels of anti-Tg were determined by chemiluminescent immunoassay (Immulite, Diagnostic Products Corporation, Los Angeles, CA, USA). Results were expressed in IU/mL, as reported in the manufacturer’s instructions.

### 4.4. Determination of Inflammatory Cytokines

Levels of interleukin-1β (IL-1β), interleukin-6 (IL-6), and tumour necrosis factor-α (TNF-α) were determined by ELISA (Bender MedSystems, Vienna, Austria). Results were expressed in pg/mL, as reported in the manufacturer’s instructions.

### 4.5. Diagnosis of Overall H. pylori Infection and CagA Status

The *H. pylori* infectious status was determined serologically using an ELISA kit with a sensitivity and specificity of approximately 96% (H pylori IgG, HpG screen ELISA kit; Genesis Diagnostics Ltd., Littleport, UK). The presence of anti-CagA IgG serum antibodies was determined using ‘CagA IgG ELISA Kit’ provided by Genesis Diagnostics, Littleport, UK (sensitivity = 96%, specificity = 97%, inter-assay coefficient of variation <12%). Levels of specific antibodies were expressed in arbitrary units (AU) per mL: individuals with 7 AU/mL of anti-*H. pylori* or anti-CagA antibodies were considered positive.

Western blotting tests were carried out to confirm *H. pylori* infection and the presence of anti-CagA antibodies [44,45]. Briefly, a whole-cell suspension of *H. pylori* CCUG 17874 (type strain), containing approximately 10^10^ bacteria, was lysed and denatured in Laemmli buffer at 100 °C for 5 min; then, proteins were resolved on 10% SDS-PAGE and transferred onto nitrocellulose. Membranes were blocked with 3% skim milk in PBS pH 7.4 containing 0.1% Triton X (PBSMT) After that, the nitrocellulose sheet was cut in strips and each strip was incubated with a different serum sample diluted 1:200. After overnight incubation at room temperature, strips were washed with PBSMT and incubated at room temperature for 90 min with an anti-human immunoglobulin G (IgG) conjugated with peroxidase, diluted 1:2000. After washings with PBSMT, the reaction was visualized by adding the substrate (H_2_O_2_ in a solution of 4-chloro-1-naphthol in Tris buffer 0.05 M pH 6.8). The reaction was blocked with water. As positive and negative controls, we used serum samples from two patients infected, respectively, by CagA+ and CagA− *H. pylori* strains and, respectively, with and without antibodies to CagA. As further controls, an anti-CagA polyclonal antiserum obtained in rabbits, kindly donated by Dr. R. Rappuoli, Novartis, Siena, was used.

### 4.6. Alignment (Bioinformatics)

We also explored the possibility that molecular mimicry phenomena could contribute to the development of autoimmune thyroid disorders. In the presence of a homology between thyroid and *H. pylori* antigens, we can hypothesize that cellular and humoral immune response against *H. pylori* infection could also affect and damage human cells.

To find regions of similarity, we “blasted” the amino acid sequences of human Tg, TPO, and TSH-R with proteins of *Helicobacter pylori* G27 (taxid:563041) (a strain isolated by one of our group, NF, in the same region inhabited by the examined individuals). The aligned human proteins were the following:

- Thyroglobulin, 2767 aa protein, GenBank: CAA29104.1
- Thyroid peroxidase, 933 aa protein, GenBank: AAA61217.2
- Thyroid stimulating hormone receptor, 764 aa protein, GenBank: EAW81333.1

Alignments were carried out using the protein databases at the National Center for Biotechnology Information (NCBI; Bethesda, MD, USA; http://www.ncbi.nlm.nih.gov/) [46] and the PHI-BLAST (Pattern Hit Initiated-BLAST) algorithm. PHI-BLAST enables to investigate protein sequences using a combination of pattern matching and local alignments to reduce the probability of false positives. Even though the minimum number of amino acids in a row to form an epitope is five, we also included longer sequences—even if the alignment was interrupted by one or two non-matching amino acids—because the probability that two proteins are similar and are effectively recognized by antibodies, increases with the length of their linear structure.

### 4.7. Statistical Analysis

The Fisher’s exact test was used to calculate the statistical differences of *H. pylori* infection and anti-CagA antibody prevalence and Odds Ratio (OR) with 95% confidence interval (95% CI) was calculate for the risk evaluation. Kolmogorov-Smirnov test and Levene test were used, respectively, to check the normal distribution and to verify the homogeneity of variances of all groups of continues variables. When these conditions were not respected, instead of the t-test, we used the Mann–Whitney test to calculate the statistical differences between groups. The results were expressed as median and interquartile range (IQR: 75–25° centile). *p* < 0.05 was considered statistically significant. Analyses were performed using the statistic software SPSS 13 (SPSS Inc., Chicago, IL, USA).

## 5. Conclusions

The different clinical forms of AITDs in relation to the CagA expression by *H. pylori* have never been exhaustively examined at the same time. Moreover, very few studies examined the relationship between CagA and clinically overt thyroid damage. The results of the present study indicate that *H. pylori* infection, particularly if caused by organisms expressing CagA, is strictly associated with GD and HT. The finding that the bacterial infection in AT patients had a prevalence comparable to that observed in controls suggests that it is important to define the clinical form of autoimmune thyroid disorder when studies on the responsibility of infections on AITD development are carried out. Another factor, i.e., the characterization of the CagA status, should be considered to avoid that results of surveys on the role of *H. pylori* infection in extra-digestive inflammatory diseases be incomplete or partial. In our work, CagA was the main determinant responsible for the increased levels of thyroid autoantibodies and inflammatory cytokines. Both factors may trigger and/or worsen the clinical presentation of the disease. Thus, it is mandatory to determine the presence of serum antibody against this antigen in patients as well as in controls.

## Figures and Tables

**Table 1:** Prevalence of overall *H. pylori* (HP) infection (%) and CagA status (%) in autoimmune thyroid diseases (AITDs) patients and controls. HP+, infected individuals; CagA+ or CagA−, infected individuals with or without anti-CagA antibodies; GD, Grave’s disease; HT, Hashimoto’s thyroiditis; AT, aspecific thyroiditis.

*H. pylori*/CagA Status	GD (%)	HT (%)	AT (%)	Controls (%)
HP+	26 (66.6)	49 (64.4)	15 (34.0)	40 (29.4)
HP−	13 (33.3)	27 (35.5)	29 (65.9)	96 (70.5)
Total	39	76	44	136
HP+/CagA+	12 (46.1)	23 (46.9)	7 (46.6)	8 (20.0)
HP+/CagA−	14 (53.8)	26 (53.0)	8 (53.3)	32 (80.0)

**Table 2:** Fisher’s exact test *p*-value and odds ratio (OR) for determining prevalence of overall *H. pylori* and CagA-positive *H. pylori* infection in patients with AITDs and controls.

**HP+ vs. HP−**	**Significance (*p*)**	**OR (95% CI)**
GD vs. Controls	*p* < 0.001	4.8 (2.32–9.95)
HT vs. Controls	*p* < 0.001	4.36 (2.43–7.8)
GD vs. AT	*p* < 0.01	3.87 (1.57–9.51)
HT vs. AT	*p* < 0.001	3.51 (1.63–7.57)
**HP+/CagA+ vs. HP+/CagA−**	**Significance (*p*)**	**OR (95% CI)**
GD vs. Controls	*p* < 0.05	3.43 (1.17–10.07)
HT vs. Controls	*p* < 0.01	3.54 (1.38–9.05)

**Table 3:** Most significantly aligning sequences of each of the three human proteins tested for structural homology with *H. pylori* G27. “Expectation value” is the number of hits one would expect to occur merely by chance. “% of identity” represents the percentage of identical amino acids in the two compared sequences”. “% of positivity” means the percentage of residues in the two aligned sequences that are very similar to each other. “% of gaps” mean the percentage of intervals lacking linear homology between two homologue sequences.

Human Proteins Aligned	Most Significantly Aligning Bacterial Proteins	Expectation Value	% of Identity	% of Positivity	% of Gaps
Thyroglobulin	Toxin outer membrane protein (Sequence ID: WP_000713656.1)	4.4	29	45	18
Thyroid peroxidase	Pyridoxine 5′-phosphate synthase (sequence ID: WP_001210902.1)	1.5	39	64	0
Thyroid stimulating hormone receptor	NADH dehydrogenase subunit L (Sequence ID: WP_001200218.1)	0.34	34	50	3
